# Origin of Ovariotomy; with an Account of the Life and Services of the Late Dr. Ephraim McDowell

**Published:** 1860-11

**Authors:** 


ART. I.—*Origin of Ovariotomy; with an account of the Life and Services of the late Dr. Ephraim McDowell, of Kentucky.* By the SENIOR EDITOR.

As ovariotomy still engrosses the attention of the medical profession in all parts of the civilized world, I am induced to believe that the following article, read before the Kentucky State Medical Society, at its annual meeting at Louisville, October, 1852, will be perused with great interest by every one who is alive to the progress of American surgery. There is the more reason for its insertion at this time in the REVIEW, because nearly the whole edition of the Transactions of the Society in which it originally appeared was destroyed by fire soon after its publication. So far as my information extends, I was the first to establish, upon a firm and indisputable basis, the claims of Dr. McDowell to the credit of having originated and first performed the operation in question; and I am, therefore, not a little solicitous that a knowledge of these claims should be more widely disseminated, both in this country and in Europe, especially in Great Britain, where the exploits of the Kentucky surgeon seem to be so little known that even his name is seldom correctly spelled.

To Kentucky belongs the honor of having furnished to the world the first case of extirpation of the ovary, for organic disease of this organ. This honor is justly and exclusively due to the late Dr. Ephraim McDowell, of Danville. From a paper published by this gentleman in the seventh volume of the Philadelphia *Eclectic Repertory,* it appears that his first operation was performed in December, 1809. It is not known, with any degree of certainty, how often Dr. McDowell repeated this operation; his published cases amount only to five, but there is reason to believe, from what I have learned from his nephew, Dr. William A. McDowell, that he performed it not less than thirteen times.

During the progress of my labors, as chairman of the committee on Surgery of the Kentucky State Medical Society, I became acquainted, in consequence of letters addressed to various gentlemen in Kentucky, Ohio, and Tennessee, with the particulars of three cases more, which, added to those published by Dr. Ephraim McDowell himself, increase the aggregate to eight. It is to be deeply regretted that Dr. McDowell did not keep a record of his operations, or communicate the results to his professional brethren. Such a contribution would not only have greatly enhanced his own reputation as a bold and original surgeon, but it would have conferred an inestimable boon upon suffering humanity.

As the operations in question reflect the highest credit, not only upon Kentucky and upon Kentucky's illustrious surgeon, but upon the United States, and as they have been mainly, if not exclusively, instrumental in directing the attention of the profession, both in America and in Europe, to a subject which has, of late years, elicited so much interest, research, and skill, it is proper that I should give a brief analysis of them. Dr. McDowell's first three cases are published in the seventh, and the last two in the ninth volume of the Philadelphia *Eclectic Repertory*.

Dr. McDowell's *first operation* was performed upon Mrs. Crawford, of Kentucky, in December, 1809. The tumor inclined more to one side than the other, and was so large as to induce her professional attendant to believe that she was in the last stage of pregnancy. She was affected with pains similar to those of labor, from which she could find no relief. The wound was made on the left side of the median line, some distance from the outer edge of the straight muscle, and was nine inches in length. As soon as the incision was completed, the intestines rushed out upon the table; and so completely was the abdomen filled by the tumor that they could not be replaced during the operation, which was finished in twenty-five minutes. In consequence of its great bulk, Dr. McDowell was obliged to puncture it before it could be removed; he then threw a ligature round the Fallopian tube, near the uterus, and cut through the attachments of the morbid growth. The sac weighed seven pounds and a half, and contained fifteen pounds of a turbid, gelatinous-looking substance. The edges of the wound being brought together by the interrupted suture and adhesive strips, the woman was placed in bed and put upon the antiphlogistic regimen. "In five days," says Dr. McDowell, "I visited her, and, much to my astonishment, found her engaged in making up her bed. I gave her particular caution for the future; and in twenty-five days she returned home in good health, which she continues to enjoy."

It will not be uninteresting here to state, that Mrs. Crawford, at the time of the operation performed upon her by Dr. McDowell, lived in Green County, Kentucky, from whence she removed, some time afterwards, to a settlement on the Wabash River in Indiana, where she died, March 30, 1841, in the seventy-ninth year of her age. There was no return of her disease, and she generally enjoyed excellent health up to the period of her death. She had no issue after the operation. Her youngest child, Mr. Thomas H. Crawford, who has kindly communicated to me these facts, was born in 1803, nearly six years before the operation.

The *second case* was that of a negress. The tumor is stated to have been very large, and so firmly adherent to the bladder and uterus as to render any attempt at extraction perfectly futile. The operator, therefore, contented himself with making a free incision into it with a scalpel, to let out its contents, which were of a thick, ropy and gelatinous character. The incision was of the same length, and made in the same situation as in the preceding case. Upwards of a quart of blood was lost in the operation. The wound, which was dressed in the ordinary manner, healed without any untoward symptom. The woman remained well for nearly five years, when the tumor began to increase again, and in twelve months it was as large as before the operation.

The *third operation* was performed in May, 1816. The subject was a negro woman; and the ovary, which was much enlarged, could be easily moved from side to side, to the left of which, however, it was adherent. Dr. McDowell made an incision into the linea alba, from an inch below the umbilicus to within an inch of the pubes, and then extended the opening toward the right side, about two inches above the former point, to afford himself more room. He next passed a ligature round the Fallopian tube, and "turned out" the left ovary, which was found to be in a scirrhous condition, and to weigh six pounds. The wound was dressed as in the preceding cases, and the woman was well in two weeks, though the ligature did not come away under five weeks. No mention is made of the manner in which the adhesions were overcome.

Dr. McDowell performed his *fourth operation* in April, 1817, upon a colored woman from Garrard County, Kentucky; removing a scirrhous ovary, weighing five pounds. The incision was made near the linea alba, but its extent is not mentioned. The ligature slipped from the Fallopian tube, after its division, and, in consequence, a great loss of blood took place. Several arteries were then tied; but this not arresting the hemorrhage, a large ligature was passed round the whole stump of the tube, and secured in the most careful manner. Although the woman was much exhausted, she happily recovery, but did not fully regain her health. "This, though the smallest ovarium I have ever extracted," says Dr. McDowell, "was much more troublesome to the patient than in any previous case. Besides experiencing severe lancinating pains in the parts, she was seldom able to discharge her urine without getting almost on her head, in consequence of the tumor falling down into the pelvis, and compressing the urethra."

His *fifth* recorded operation was performed on the 11th of May, 1819. The patient, likewise a negress, and the mother of one child, was from Lincoln County, Kentucky, and was supposed by her physician to be laboring under ascites, as the tumor was very large and fluctuating. After having given her hydragogue medicines for some time without any benefit, Dr. McDowell tapped her, and drew off thirteen quarts of thick, gelatinous fluid. The operation was repeated in two months, and it was now ascertained, after the matter was all evacuated, that there was a firm substance, of considerable size, which was evidently a dropsical ovary. Some months after this she was again tapped, and the opening was enlarged so as to admit the finger, which was freely used as a probe, that there might no longer be any doubt respecting the true character of the disease. The incision was made on the left side of the median line, down to the tumor, which was found firmly adherent to the parietes of the abdomen and to the intestines by slender cords, which were easily separated by the hands. The ligamentous bands, attaching the tumor to the uterus, were surrounded by ligatures, after which the tumor was opened, its contents discharged, and the sac extracted. The fluid measured sixteen quarts, and was of a gelatinous character, intermixed with a considerable quantity of hair, and a body resembling very much, in shape, the front tooth of a cow. Violent peritonitis ensued, followed by death on the third day. The uterus and right ovary were perfectly natural, and the ligatures were well applied, and not in a situation likely to injure the adjoining parts.

Dr. McDowell was assisted in this operation by his nephew, Dr. W. A. McDowell, and it was performed in the presence of Drs. Weissiger, Tomlinson and Horr.

It will thus be perceived that of these five cases, three were entirely successful; that one recovered, and remained well for nearly five years, when the tumor recommenced growing; and that one died from peritoneal inflammation within three days after the extirpation of the diseased mass.

A highly respectable lady, Mrs. Overton, aged fifty-five years, of the neighborhood of Nashville, Tennessee, consulted Dr. McDowell, through her professional adviser, Dr. James Overton, in July, 1822, concerning a tumor in the left side of the abdomen, which she had first noticed some time during the previous December. Being a member of a family inclined to corpulency, she paid no particular attention to it for several months, the more especially as it was free from pain and soreness. The enlargement of the abdomen continued to increase gradually, and early in the following May she felt distinctly, on the left side and a little below the level of the umbilicus, a small globular tumor, destitute of sensibility, and movable from side to side, as well as from above downward. About the middle of June, by which time the swelling had considerably augmented in volume, the patient was seized with pains in the back, hips, and thighs, much resembling the first pains of parturition. By the use of laxatives, warm bathing, and anodynes, these symptoms were subdued, and she enjoyed an interval of ease and health, until the latter part of July, when there was a recurrence of the local distress, in a more aggravated form, with great tenderness on pressure. The urinary secretion was natural, both as to quantity and quality, and the uterus appeared to be perfectly sound. No fluctuation could be discovered at this time in the swelling; and the integuments of the abdomen were quite lax, except at the site of the enlargement, where they were very tense.

When Dr. DcDowell visited the patient, in the summer of 1822, the tumor filled nearly the whole of the abdomen, and she had the appearance of a female in the sixth month of utero-gestation. The general health was a good deal impaired, from fever and want of sleep, and there was a sense of weight and dragging in the pelvis, with acute pain in the tumor, perinæum, and thighs.

Supposing the disease to consist in a morbid enlargement of the left ovary, Dr. McDowell designed to extirpate it with the scalpel, and for this purpose made an incision, from five to six inches in length, along the linea alba, over the most prominent part of the tumor down to the peritoneum. Having laid bare this membrane, he proceeded cautiously to divide it, intending to make an opening sufficiently large to admit of the removal of the diseased organ. In this, however, he was disappointed; for he had no sooner made his first incision through the peritoneum than there gushed out, in a full stream, a bloody-looking serum, which continued to flow till the sac which had contained it was apparently entirely empty. The quantity thus lost was about one gallon. The edges of the wound were then approximated by several interrupted sutures, light dressings were applied, and the abdomen was encircled by a broad bandage. This constituted the whole of the operative procedure. No attempt was made, or even deemed practicable, to extirpate the diseased organ, inasmuch as it adhered so closely to the perionteum as to render it impossible to distinguish or separate it. Indeed, Dr. McDowell supposed that he was dividing the peritoneum only when the knife penetrated the ovarian sac. The circumstance took him, as well as every one present, by surprise, because it was entirely unanticipated.

The wound continued to discharge matter for some time after the operation, from the lower extremity of the incision, where a tent was kept for that object. The fluid gradually lost its sanious character, and as it diminished in quantity it assumed more and more the appearance of healthy pus. The wound was entirely healed at the end of about five weeks; and the patient, who lived from fifteen to twenty years after the operation, enjoyed excellent health; nor did she, at any subsequent period, suffer any pain or uneasiness which could be justly ascribed to disease of the ovary, or any other organ connected with the uterus.

The interest of this case is heightened by the circumstance that the late President Jackson, who was a near neighbor of the patient, was present at the operation, assisting in holding her hands, and encouraging her resolution.

For the above interesting and valuable details, I am indebted to Dr. James Overton, an eminent practitioner of Nashville, Tennessee, who was present at the operation, and who had charge of the case, both before and after the operation by Dr. McDowell.

For the details of the next case, I am indebted to the late Dr. Galt, of Louisville.

The subject of this case was Miss Plasters, of Kentucky, who was attacked, in the winter of 1821, with enlargement and pain of the right ovary. The disease gradually increased, and in February, 1823, she was tapped for the removal of the contents of the tumor. The dropsical symptoms, however, soon reappeared, and believing that excision of the affected organ afforded the only chance of permanent relief, her medical advisers, Dr. Galt and Dr. Ragland, requested her to consult Dr. McDowell. I have not been able to obtain any information as to the age of the patient and the size of the tumor; but from a letter written by Dr. McDowell to Dr. Galt, some time after the operation, I learn that the enlarged viscus filled the entire abdominal cavity, and that out of nine cases that had presented themselves with this disease, up to the period adverted to, that of Miss Plasters appeared by far the most hopeless. Upon her arrival at Danville, she was so extremely debilitated that it was believed she would hardly be able to sustain the shock of the operation.

The patient having undergone the necessary preliminary treatment, the operation was performed on the 12th of May, 1823. An incision was made into the abdominal cavity, extending the whole length of the linea alba. Finding the tumor so large that it could not be removed entire, a free opening was made into it, discharging about six pints of fluid. The morbid mass was then lifted from its bed, though not without difficulty, a ligature having previously been cast round its foot-stalk or uterine attachment. The abdominal cavity having been cleared of blood and water, the edges of the wound were carefully closed in the usual manner, and the woman put to bed.

The omentum is said to have been much inflamed and thickened, not, as Dr. McDowell supposed, from the effects of the previous tapping, but from organic disease of its own structure. For ten or fifteen days after the operation, there was a bloody, putrid discharge from the wound, "which," says Dr. McDowell, "I am well assured could arise from nothing but sloughing of the omentum."

Nothwithstanding her debilitated condition before and for some time after the operation, Miss Plasters entirely recovered. On the fourth of August, less than three months after the removal of the tumor, Dr. McDowell informed Dr. Galt that she was in "perfect health and spirits."

In April, 1824, the health of Miss Plasters was so good that she engaged herself to marry in the spring. About this period, however, dropsy of the abdomen set in, rendering it necessary to tap her, and after that Dr. Galt lost sight of her. How long she survived he did not know.

In commenting upon this case, in the letter already referred to, Dr. McDowell holds the following language : "This case proves that appearances in surgery are often deceitful, and that while the taper of life continues to burn, although it be faint, there is yet hope; for Miss Plasters has certainly disappointed most of her friends and all that saw her. My own hopes, at times, were but faint."

"How it is," he continues, "that I have been so peculiarly fortunate with my patients of this description, I know not; for, from all the information I can obtain, there has not one individual survived who has been operated on elsewhere, for diseased ovaria. I can only say that the blessing of God has rested on my efforts." Miss Plasters, it will be remembered, was Dr. McDowell's ninth case.

For the particulars of the following case, in which the operation of ovariotomy was attempted, but not successfully performed, I am indebted to the kindness of the late Professor Drake, of Cincinnati, who saw the patient both before and after she fell into the hands of Dr. McDowell, and who kept full notes of the symptoms and other circumstances. The operation was witnessed by the husband of the lady, a very intelligent gentleman, who afterwards gave an account of it, in a letter to Professor Drake, which I have carefully perused.

Mrs. Delano, aged thirty-eight, a native of Kentucky, but now a resident of Chillicothe, Ohio, has been married eight years, but never had any children. She is of a bilious temperament, has always menstruated regularly, and was never sick until five years ago. In the autumn of 1822, she began to be conscious of a fullness in the abdomen, between the hips and ribs, but this was so slight as to escape observation. For a year the local disease made no apparent progress, and her general health was good. In 1823–4–5, she suffered, at intervals, from nausea, colic, and attacks of fever. In December of the latter year, the colic became constant, and there was great soreness in the sides and limbs. A hard tumor was discovered, about this time, in the right ileo-hypogastric region, which has steadily increased in volume, and now occupies the whole abdominal cavity, from the pubic symphysis to above the umbilicus, reaching outwardly as far as the costal cartilages. It is hard, irregular, slightly movable, and cannot be traced under the ribs. It is somewhat sore on pressure, as well as painful, though less so than formerly. The bowels are usually regular; she lies best on her right side; and she has frequent vomiting, with some cough.

The above memorandum was made, in substance, by Professor Drake, on the 24th of October, 1826; immediately after which Mrs. Delano went to Danville, to consult Dr. McDowell about the propriety of an operation. In the interview which ensued between the parties, "I learned from Dr. McDowell," writes the husband of the lady, "that he had operated for diseased ovaria in nine or ten cases, in eight of which he had been successful. Some of these, I believe, were of that character termed dropsy of the ovary, the tumor consisting of a sac, inclosing a quantity of liquid, gelatinous matter; in the other cases the tumor was solid, and appeared to be composed of a gristly, cartilaginous substance. In every instance attended with success, the tumor was loose and floating, except in one or two cases, where it was filled with water, which was drawn off, the sac being suffered to remain. In every other case, where the tumor adhered, the attempt to operate was unsuccessful; or, if Dr. McDowell succeeded in extracting the morbid growth, as he probably did in one instance, the patient died." Such, then, appeared to be the information obtained from Dr. McDowell.

I have given the above extract from Mr. Delano's letter, because it shows how often, and with what success, Dr. McDowell had performed this operation, at the time the writer consulted him about the case of his wife. His first case occurred, it will be recollected, in 1809, that is, seventeen years previously to the one under consideration.

From the difficulty, or rather impracticability, of moving the tumor about, Dr. McDowell naturally concluded that it must be extensively adherent. Believing, however, that extirpation was not impossible, and the lady being anxious to be relieved, an operation was determined upon. An incision was accordingly made, in the usual manner, through the linea alba, and the tumor exposed by separating the peritoneum and omentum, by which it was enveloped. The patient, at this stage of the operation, became so rapidly and excessively debilitated, that apprehensions were entertained for her immediate safety. The tumor, as had been previously conjectured, was found to be extensively and strongly adherent, and its extraction was pronounced impracticable. In about two weeks the wound was healed, without any material change in the general health.

Dr. Drake visited Mrs. Delano on the 11th of March, 1827, about four months and a half after the operation, and found her excessively emaciated, with swelling of the right leg, and all the symptoms of gradual exhaustion. A cicatrix, from the operation, existed in the linea alba, and was about two inches long. The whole abdomen was excessively protuberant, from the pubic symphysis to the ensiform cartilage. The liver, very hard and greatly enlarged, bulged out high between the short ribs and the umbilicus, over into the left side. Here, in contact with the ovarian tumor, in a kind of groove between them, the colon passed across below the navel. At the centre of the colon was a small, flattish tumor, which felt very hard, and could be moved along the bowel down into the sigmoid flexure. The ovarian tumor filled the right iliac region, and extended, in front, nearly to the umbilicus, crossing the linea alba, and being, seemingly, more prominent on the left side than on the right. It was fixed during respiration, and appeared to be immovable. The liver, on the other hand, obeyed the motions of the diaphragm. Her menses had ceased only about three months before. Death occurred soon after Dr. Drake's visit; no examination of the body was made.

Dr. McDowell was born in Rockbridge County, Virginia, on the 11th of November, 1771. His father, Mr. Samuel McDowell, was for many years a member of the legislature of that State, and appears to have been a man of great popularity in his district. In 1782 he was appointed, along with Mr. Caleb Wallace and Dr. Flemming, by the legislature of Virginia, a commissioner to settle the land-claims of the Territory of Kentucky, and entered immediately afterwards upon the discharge of the duties of his onerous and responsible office.

The following year he removed with his family to Kentucky, and settled near Danville, where he lived till the time of his death, in August, 1817. During his residence here he was appointed Judge of the District Court of Kentucky, and, along with his associates, Judge Wallace and Judge Mütter, assisted in organizing the first court at Danville, which was also the first court ever formed in the district or territory of Kentucky. He remained upon the bench until within a few years of his death.

In January, 1755, Judge McDowell married Miss McClung, of Virginia. The result of this union was twelve children, of whom Ephraim, the subject of this sketch, was the ninth, and the only survivor of whom is Colonel Joseph McDowell, of Danville.

When scarcely two years of age, Ephraim McDowell was brought by his parents to Kentucky. It is not known when he began to go to school; but we are told that he was educated by two gentlemen of the name of Worley and James, who conducted a classical seminary, first at Georgetown, and afterwards at Bardstown. What proficiency he made under the tuition of these teachers, or how long he remained under their charge, I am not informed. The probability, however, is that he never made any great progress in his classical studies, although it would seem that he had some fondness for them. The few brief articles which he contributed to the medical journals, after he had attained to professional eminence, clearly indicate that his early education was defective.

Soon after leaving school, he entered upon the study of medicine, his preceptor being Dr. Humphreys, of Staunton, Virginia, a graduate of the University of Edinburgh. With him he read from two to three years, when, without having, so far as I can learn, attended any lectures in Philadelphia, at that period the only seat of medical education in the United States, he went to Scotland, to avail himself of the facilities afforded by the celebrated school at the capital of that country. He was a member of the medical classes of the University of Edinburgh, for the sessions of 1793 and 1794. This institution was at that time at the very zenith of its renown, attracting pupils from all parts of the civilized world, and overshadowing every other medical school in Europe. We may well imagine with what interest and delight the young and ardent Kentuckian, thirsting after knowledge, drank in the waters of science as they gushed forth from the eloquent lips of a Gregory, a Black, and a Monro, men whose fame was upon the tongue of every medical student and physician in Europe and America. We may suppose, too, that, amid the novel scenes which surrounded him, his mind often reverted to his native country, lamenting its deficiencies for the acquisition of a sound medical education, and regretting, perhaps in bitter terms, the time which he had spent, to so little purpose, in the office of his Staunton preceptor. We may imagine, moreover, that one just fresh from the wilds of America must have felt no little restraint, and even embarrassment, in the polished and refined circle of students in the modern Athens. His principal friend and companion, during his attendance upon the Edinburgh school, was Dr. Samuel Brown, afterwards the elegant and accomplished Professor of the Theory and Practice of Medicine in Transylvania University, and brother of the Hon. James Brown, our Minister at the Court of France. Of his mode of living and his habits of study, while at this far-famed institution, unfortunately nothing is known. His nephew, Dr. William A. McDowell, who was intimately acquainted with his whole history, states that the surgical lectures at the University were not satisfactory to him, and that, in consequence, in his second year he took a ticket to the private course of the celebrated Mr. John Bell. I am not certain who delivered the surgical lectures at that period in the University; probably they were given jointly by Dr. Monro, the second, and Dr. Russell, the latter of whom was always regarded as a very dull teacher by his classes; while the former, although more sprightly and animated, was very prosy in comparison with John Bell, whose enthusiasm and ardor were absolutely boundless.

It is difficult to conceive, at this distant day, the charm which this great teacher infused into his subject, and the ambition which he inspired in his pupils. All loved him; many worshiped him; not a few idolized him. Among the latter was the subject of this memoir. During his attendance upon his prelections, the young American was enraptured by the eloquence of his teacher; and the lessons which he imbibed, while thus occupied, were not lost upon him after his return to his native country. Mr. Bell is said to have dwelt with peculiar force and pathos upon the organic diseases of the ovaries, speaking of their hopeless character, when left to themselves, and of the possibility, nay practicability, of removing them by operation. The instruction thus given made a powerful impression upon Dr. McDowell, which, as has been already stated, was not lost upon him after he took leave of the academic groves of Edinburgh.

I am not able to say, from the facts before me, whether Dr. McDowell was graduated at Edinburgh or not. His brother, Colonel McDowell, states that he was, and in this opinion he is joined by his son, as well as by his nephew, Dr. William A. McDowell. My belief that he did not take a degree in Scotland is based upon three facts: first, that no diploma of the kind has been found among his papers; secondly, that Dr. Samuel Brown, his classmate at Edinburgh, solicited him, after he had attained to eminence in his profession, to permit him and his friends to draw up an account of his cases of ovariotomy, in Latin, in order that it might be sent to Edinburgh, to secure him a degree; and thirdly, that in 1825, the University of Maryland conferred upon him the honorary degree of Doctor of Medicine: a circumstance which would hardly have happened had he been a regular graduate. Be this as it may, the fact that he was or was not a graduate ought not, in the slightest manner, to derogate from his character as a professional man, especially when it is recollected how much more difficult it was then than it is now to obtain a degree in medical schools.

After a residence abroad of about two years, during which he stored his mind with a large amount of valuable information, Dr. McDowell returned to Kentucky, in 1795, and settled at Danville, the scene of his future labors. He immediately entered upon his professional career, nor was he slow in acquiring business. The fame of his foreign tour had preceded him, and served to introduce him into practice. It was known that he had been a student of John Bell, one of the most celebrated surgeons of the age, and that he had devoted himself, with special assiduity, to the study of anatomy and surgery during his sojourn in Scotland. The consequence was that patients soon flocked to him, at first from the neighborhood, and subsequently from all parts of the Southwest, for his aid and advice. Those who were unable, on account of the peculiarity of their ailments, or the roughness of the roads, to come to him, he visited at their own homes, often remaining with them several days, or, when the case was unusually urgent, even a week or two. All the important operations that were required for hundreds of miles around were performed, for a number of years, exclusively by him. At that time he was almost the sole occupant of the field of surgery in the West. Dr. Dudley, since so celebrated for his surgical exploits, had not yet commenced his professional studies, and none of the larger towns of Kentucky had any surgeons of distinction, or even ordinary capacity. The only exception, probably, to this statement, was Bardstown, the residence of Dr. Brashear, who, early in the present century, performed the first successful amputation at the hip-joint in the United States, and who enjoyed for some time considerable reputation as an operator in Nelson and the adjacent counties. Dr. Brashear, however, was scarcely a competitor of Dr. McDowell, or if he was at any time, he did not long continue to be; for soon after the achievement above alluded to, and while he was still a very young man, he abandoned his profession, and moved to Louisiana, where he engaged in planting, even then a lucrative and respectable pursuit. Cincinnati, too, was at that time, and indeed for a long period afterwards, without a surgeon of any respectability or eminence. Thus, as I have already stated, Dr. McDowell was, for a number of years, in the undisputed possession of the surgical field, not only of Kentucky, but of the entire Southwest. How well he cultivated this field, what honor he conferred upon his adopted State and upon the country generally, is too well known to require any comment.

In 1802, in the thirty-first year of his age, Dr. McDowell married Sarah Shelby, a young lady of great personal beauty and excellence, daughter of Governor Shelby, one of the most distinguished citizens of Kentucky. The result of this alliance, which greatly augmented his happiness, was eight children, only three of whom survive. Mrs. McDowell died at Danville only about ten years ago.

Dr. McDowell had practiced medicine and surgery for fourteen years, and had secured for himself a large share of reputation for his bold and successful exploits, when, in the autumn of 1809, he was consulted by Mrs. Crawford, the subject of a large ovarian tumor, whose case, from its novelty and the attendant circumstances, must forever remain memorable in the annals of our profession. After a most thorough and critical examination, Dr. McDowell informed his patient, a woman of unusual courage and strength of mind, that the only chance for her relief was excision of the diseased mass. He explained to her, with great clearness and fidelity, the nature and hazard of the operation; he told her that he had never performed such an operation, but that he was ready, if she were willing, to undertake it, and to risk his reputation upon the issue, adding that it was an experiment, but an experiment well worthy of trial. Mrs. Crawford listened to the surgeon with great patience and coolness, and at the close of the interview promptly assured him that she was not only willing, but anxious to submit to his decision; asserting that any mode of death, suicide excepted, was preferable to the ceaseless agony she was enduring, and that she would hazard anything that held out even the most remote prospect of relief. The result has been long before the profession. Mrs. Crawford submitted to the operation, and thus became the first subject of ovariotomy of whom we have any knowledge.

It has already been seen that Dr. McDowell, during his residence at Edinburgh, attended the surgical lectures of Mr. John Bell, who took especial pains to direct the attention of his pupils to the diseases of the ovaries. It is not improbable that the young Kentuckian, while listening to the teaching of the ardent and enthusiastic Scotchman, determined in his own mind to extirpate these organs in the first case that should present itself to him after his return to his native country. The subject had evidently made a strong impression upon him, and had frequently engaged his attention and reflection. He had thoroughly studied the relations of the pelvic viscera, in their healthy and diseased conditions, and felt fully persuaded of the practicability of removing enlarged ovaries by a large incision through the wall of the abdomen. He knew very well that the Cæsarean section had been repeatedly performed with success, and he could perceive no reason why ovariotomy should be attended with more difficulty to the surgeon, or greater hazard to the patient.

When Dr. McDowell undertook this operation, he was not aware that it had ever been performed by any one else, a precedence which certain writers have attempted to prove. In speaking of his first case, he distinctly states that he had "never seen so large a substance extracted, nor heard of an attempt or success attending any operation, such as this required."* Nor was such an operation ever performed before. From all the testimony that I have been able to collect upon the subject, I am satisfied that it was first executed by Dr. McDowell.

Until I had carefully investigated this matter, I was of the opinion, in common with many others, both in this country and in Europe, that a foreign surgeon, L'Aumonier, of Rouen, had anticipated our countryman in this bold and daring undertaking. The attempt to remove this organ is said to have been made by this gentleman as early as 1776. Upon inquiry, however, I find that this was not the fact, but that the case upon which he operated was one merely of abscess of the ovary, consequent upon parturition. There was, of course, no necessity here for extirpation; all that was done by L'Aumonier was to puncture the abscess, to give vent to its contents. He never dreamed of excising the organ, and

* Eclectic Repertory, vol. vii. p. 242. Philadelphia, 1817.

it is surprising that Dr. John Mason Good and other learned authors should ever have referred to the case as one of ovariotomy.

Equally unfounded, in this respect, are the claims of Professor Dzondi and of Professor Galenzowski, whose names precede that of Dr. McDowell, in the elaborate and valuable table of ovariotomy, by Dr. Washington L. Atlee, of Philadelphia. In a work published by the former of these writers, at Halle, in 1816, entitled *Beiträge zur Vervollkommung der Heilkunde*, or, Contributions toward the Improvement of the Healing Art, is an account of a pelvic tumor, in which a cure was effected by drawing out the cyst through an incision in the wall of the abdomen, and, after inducing mortification in it by means of long tents, extracting it piecemeal with a pair of broad forceps.* The tumor thus treated, however, was not ovarian, nor was the patient a female, but a lad, twelve years of age, of the name of Christopher Shultz, who had a circumscribed tumor as large as his head in the hypogastric region. Dzondi relates other examples of a similar nature, relieved by the same mode of management, and he expresses the opinion that this operation might be resorted to with equal success in ovarian dropsy, provided the sac is situated superficially, and is not affected with ulceration or scirrhus. It is difficult to conceive how such a case could ever have been adduced as one of extirpation of the ovary, the more so, when it is recollected that the author himself never advanced such a claim.

The case of Galenzowski, of Wilna, was also one very different from extirpation of the ovary. But this is not all; his operation was not performed until March, 1827, eighteen years after Dr. McDowell's now celebrated operation upon Mrs. Crawford. The tumor, in the case in question, was multilocular, of large size, and so firmly and universally adherent to the posterior wall of the abdomen as to render its total extirpation impracticable. Galenzowski, therefore, made a large incision into its cavity, according to the method of Le Dran; and then, passing his fingers into it, he tore up its cells, evacuated its contents, and secured the sac with a ligature to the external wound, to prevent the possibility of peritoneal effusion. On the thirty-second day a piece of the sac was found in the dressings; another was discharged on the fifty-second day; and, finally, a third on the sixty-second. The patient was discharged on the seventieth day, having only a small fistule in the hypogastric region.

The particulars of Galenzowski's case are contained in the twelfth volume of Graefe & Walther's *Journal der Chirurgie;* and in a translation of his paper, originally published in Latin, in the ninth volume of the North American Medical and Surgical Journal, issued at Philadelphia,

---

* American Medical Recorder, vol. iii. p. 62.

in 1830. An abstract of it may be found in Malgaigne's Operative Surgery, by Brittan, p. 391, Philadelphia, 1851; and in the *Dictionnaire de Médecine*, tome xxii. p. 592, Paris, 1840.

In consequence of the novelty of Dr. McDowell's operations, and of the loose manner in which they were drawn up for publication, an attempt was made by certain writers, both in this country and in Europe, to deny their authenticity, and to cast discredit upon the author's veracity. Among the various detractors who busied themselves in this way, no one was more loud and clamorous than Dr. James Johnson,* the editor of the London Medico-Chirurgical Review, a periodical well known in the United States. In speaking of Dr. McDowell's first case, he remarks: "Dr. Mac visited the patient at the end of five days, though she had come to his own residence to have the operation performed! He found her engaged in making her bed! She soon returned to her native place quite well. *Credat Judæus, non ego.*" In adverting to the second case, the reviewer says: "We cannot bring ourselves to credit the statement." It is proper to add that Mrs. Crawford, the subject of the first operation, performed in 1809, and so sneeringly spoken of by Dr. Johnson, survived until 1841, or until after the completion of her seventy-eighth year; and that the authenticity of the second case, concerning which he expresses so much incredulity, is equally well established.

In a subsequent article upon this subject, published in October, 1826, the same writer indulges in the following language:† "A back settlement of America, Kentucky, has beaten the mother country, nay, Europe itself, with all the boasted surgeons thereof, in the fearful and formidable operation of gastrotomy with extraction of diseased ovaria. In the second volume of this series, page 216, we adverted to the cases of Dr. McDowell, of Kentucky, published by Mr. Lizars, of Edinburgh, and expressed ourselves as skeptical respecting their authenticity. Dr. Coates, however, has now given us much more cause for wonder at the success of Dr. McDowell; for it appears that out of five cases operated on in Kentucky by Dr. McDowell, four recovered after the extraction, and only one died. There were circumstances in the narratives of some of the first three cases that raised misgivings in our minds, for which uncharitableness we ask pardon of God, and of Dr. McDowell, of Danville." Such language needs no comment; it speaks for itself, for it carries with it its own condemnation. When the learned, caustic, and ungenerous editor of the London Medico-Chirurgical Review indited it, he was ignorant—perhaps, willfully ignorant—of the fact that he was slandering the

* Eclectic Repertory, vol. vii. p. 242. Philadelphia, 1817.

† Medico-Chirurgical Review, January, 1825.

father of ovariotomy, and speaking sneeringly of a State that has given birth to the first lithotomist, and the first American statesman of the nineteenth century.

But it need not surprise us that Dr. McDowell's cases of ovariotomy should have been treated with contempt abroad, when attempts were made to discredit them at home. What effect these attempts exerted upon the professional mind of the United States, it would be useless, at this remote day, to inquire; suffice it to say that they aroused the assailed party to a sense of self-defense, and caused the publication of additional cases, confirmatory of those that had previously appeared in the Eclectic Repertory.

It would seem that Dr. McDowell kept no notes of any of his cases, and that he was prevailed upon, with the greatest difficulty, to publish an account of the first three in the Eclectic Repertory. His nephew, Dr. William A. McDowell, states that he had to urge, among other inducements, the debt of gratitude which his uncle owed to Mr. John Bell, and his obligation to compliment that celebrated surgeon with an exhibition of the exploits of his pupil, in the execution of an operation the practicability of which he had been at so much pains to teach in his lectures. This appeal had more weight in deciding Dr. McDowell than anything else that could be urged. He finally drew up an outline of his cases, referring to his ledger for his dates, and to his memory for the facts. They were not written out with sufficient minuteness and precision; but they had, at least, two capital merits—truthfulness and brevity. A copy of the paper, with a letter of acknowledgment, was forwarded to Mr. Bell, but this he never received. He was absent at the time, traveling on account of his health in Italy, whence he never returned alive. The paper subsequently fell into the hands of Mr. Lizars, who published it seven years afterwards, in the thirty-second volume of the Edinburgh Medical and Surgical Journal, in consequence of an attempt which he made to ovariotomize a female, to the peril of her life, who was found to be afflicted only with abdominal obesity.

Another copy of these cases was sent, in the autumn of 1816, to Dr. Physick, of Philadelphia, with a request that, if found worthy, he should have them published in some medical journal. It is not known whether the "Father of American Surgery" ever read the paper, or investigated the nature of the operation which it described; certain it is, he never took any notice of it, either to Dr. McDowell, his pupils, or any one else. He had no time to bestow upon the subject, or the subject was unworthy of his attention. He might have thought the author of the paper a backwoods impostor, or a man who was speaking for Buncombe.

Dr. William A. McDowell, who was bearer of the above dispatch, was more successful in his interview with Dr. James, the modest, amiable, and benevolent Professor of Midwifery, in the University of Pennsylvania. This gentleman had been in the habit of depicting to his pupils, with every revolving lecture term, and in the most harrowing language, the fatality and utter hopelessness of all attempts, past, present, and to come, to cure organic diseases of the ovaries by operation. He seized with avidity the intelligence communicated to him by the younger McDowell, and immediately requested an account of the cases for publication in the Eclectic Repertory, of which he was then one of the editors. He also read the cases to his class, who received them with the most rapturous applause.

The publication of the cases in the Eclectic Repertory elicited hardly any notice. Indeed, so far as could be ascertained, it encountered general incredulity, if not positive ridicule. The paper of Mr. Lizars, in the Edinburgh Medical and Surgical Journal, met with a better fate; for it at once attracted marked attention, and produced strong excitement throughout the medical circles of Europe; a circumstance which reacted with electrical force and rapidity upon the United States. The question of the reliability of the reported cases was at once thoroughly investigated and established; and the operation has since been repeatedly performed with success in nearly all parts of the civilized world. Ovariotomy is now one of the established resources of surgery; and for this boon our profession and mankind are indebted to Dr. Ephraim McDowell.

It is not positively known, even to his most intimate surviving friends, how often Dr. McDowell performed the operation of ovariotomy. Dr. William McDowell, who was a member of his family for nearly seven years, five as a student and two as a partner in practice, states that up to the period of his removal to Fincastle, Virginia, in 1820, his uncle had had seven cases, all, save one, successful. Six of these operations Dr. McDowell witnessed, and in two he handled the knife under Dr. Ephraim McDowell's direction. "I acted," he modestly adds, "in the capacity, as I conceived, of a sort of an amanuensis. In his first operation," continues this gentleman, "in 1809, Dr. James McDowell, who was Dr. Ephraim McDowell's nephew, partner, and brother-in-law, and a young man just commencing practice, used the knife under similar circumstances, as it respects the external incision. Dr. Ephraim McDowell had, at the time, determined to decline practice in favor of Dr. James McDowell. The death of the latter occurring shortly afterwards, changed the arrangement. Under the same circumstances, Dr. A. G. Smith, who succeeded me as a partner of Dr. McDowell, operated in two or three cases."

Dr. William A. McDowell informed me that he had reason to believe, from reliable testimony, that his uncle performed this operation altogether thirteen times, exclusive of the cases of Dr. Smith. He declared that he himself had been cognizant of two more successful cases: one, that of Mrs. Overton, of Tennessee, and the other, that of Miss Gilmore, of Pulaski County, Kentucky; making in all eight cures, respecting which there can be no doubt. Dr. McDowell's success does not seem to have been so great in his latter as in his earlier operations, but the precise ratio cannot now, unfortunately, be ascertained.

It may not be improper to state, in concluding the consideration of this part of Dr. McDowell's professional services, that an attempt was made, many years ago, to deprive him of the credit of his first operation, by ascribing the performance of it to his nephew, Dr. James McDowell, at the time his partner in practice. In consequence of this attack upon his veracity and pretensions to surgery, Dr. McDowell was induced, in 1826, to address a printed card to the "physicians and surgeons of the West, and particularly to the medical faculty and class at Lexington," in vindication of his claims. After remarking that he had visited Mrs. Crawford, the subject of the operation in question, at her residence in Green County, Kentucky, a distance of sixty miles from Danville, for the purpose of examining her case, and that she soon after came to his own house to undergo the operation of ovariotomy, he says: "My nephew, Dr. James McDowell, whom I had brought up, had graduated a few months before this time, in Philadelphia, and had commenced business as my partner. Being in delicate health at the time, it was my intention to remove to the country in the spring, or so soon as I could establish my nephew in business.

"From the time of Mrs. Crawford's arrival, he had made frequent attempts to persuade me from operating; but, finding my determination was fixed, he agreed to be present, but not until the morning I operated, and as my partner to assist; for should the patient die, the responsibility was all my own; should the patient live, it would assist him in his outset in business.

"The day having arrived, and the patient being on the table, I marked with a pen the course of the incision to be made; desiring him to make the external opening, which, in part, he did; I then took the knife and completed the operation, as stated in the Eclectic Repertory. Although the termination of this case was most flattering, yet I was more ready to attribute it to accident than to any skill or judgment of my own; but it emboldened me to undertake similar cases; and not until I had operated three times—all of which were successful—did I publish anything on the subject. I then thought it due to my own reputation and to suffering humanity to throw all the light which I possessed upon diseased ovaria."

It is not necessary that I should enter into a formal examination of the claims of Dr. McDowell as the author of the operation in Mrs. Crawford's case. The paper from which I have quoted the above extracts is accompanied by three certificates, all testifying to the truth of Dr. McDowell's statement. One of these certificates is from Mrs. Crawford herself; another from Mrs. Baker, one of her female attendants; and the third from Mr. Charles McKinny, a private pupil of Dr. McDowell, who, with Mrs. Baker, witnessed the whole proceeding. He states, expressly, that Dr. James McDowell made the external incision as directed by his uncle, and that then the latter took the knife and extracted the diseased ovary. He asserts, moreover, that he never heard Dr. James McDowell claim the credit of the operation. When we add to these facts, the statement of Dr. William A. McDowell, that he and Dr. Smith both assisted Dr. Ephraim McDowell, on several occasions, in the same manner, it follows, as a necessary corollary, that the claims set up in behalf of Dr. James McDowell, who is said to have been a young man of great professional promise, must fall to the ground as untenable.

I have endeavored to establish the claims of Dr. Ephraim McDowell to the operation in question, upon a firm and immutable basis. My only motive for so doing has been a wish to defend truth, to subserve the interests of surgical science, and to award credit where alone credit is due. How far I have succeeded in my effort let others judge.

But Dr. McDowell's claims to distinction do not, by any means, rest upon his exploits, novel and brilliant as they were, as an ovariotomist. He ranked also deservedly high as a lithotomist. For a time he was almost the only physician in Kentucky who performed this operation. In the latter period of his life he was eclipsed, in this branch of surgery, by his neighbor, Dr. Dudley, of Lexington, who, after the establishment of the Transylvania Medical School, for many years almost monopolized the stone cases in Kentucky and the adjacent States. It is not known how often Dr. McDowell performed this operation; but it is positively ascertained that he had, up to 1828, two years prior to his death, executed it thirty-two times, and that without the loss of a single patient. Such success is as rare as it is creditable to Dr. McDowell's skill and judgment. He confined himself to the lateral method, and early in life opened the bladder with the gorget, but afterwards made his deep incisions with the knife.

One of his most interesting cases, not from any peculiar circumstances or merit, but from the exalted position attained by the patient, was that of the late James K. Polk, afterwards President of the United States. This gentleman had suffered from symptoms of vesical calculus from an early period, and in his seventeenth year he was induced to visit Danville in search of advice. The operation was performed in the autumn of

1812, with Dr. McDowell's usual skill, and a happy recovery was the consequence. The calculus was of small size, very hard and heavy, with a rough, tuberculated surface. Mr. Polk carried it home with him, not in his bladder, but in his pocket, to show it to his friends and neighbors, with whom it was a source of great curiosity. In a letter, dated in Maury County, West Tennessee, December 3d, 1812, he informs his surgeon of the progress of his cure, and feelingly expresses his sense of gratitude for the services which he had received from him. This letter, as a specimen of literary composition, is far below mediocrity; it is badly spelled, and written in the worst style. In these respects, it is in striking contrast with another letter addressed to Dr. McDowell, nearly fourteen years afterwards, when Mr. Polk represented his adopted State in the Congress of the United States. In this communication, written with great accuracy, and even eloquence, Mr. Polk again expresses his gratitude to Dr. McDowell; speaks of the excellence of his health, and alludes to the manner in which he had spent his time since his recovery from the operation. "I have been enabled," he says, "to obtain an education, study the profession of the law, and embark successfully in the practice; have married a wife, and permanently settled in Tennessee; and now occupy the station in which the good wishes of my fellow-citizens have placed me. When I reflect, the contrast is great indeed, between the boy, the meager boy, with pallid cheeks, oppressed and worn down with disease, when he first presented himself to your kind notice, in Danville, nearly fourteen years ago, and the man at this day in the full enjoyment of perfect health." I take great pleasure in alluding to these letters of Mr. Polk. The career of that gentleman, and that of his surgeon, show how early obstacles may be vanquished by industry, and how perseverance enables men, from small beginnings, to attain to great ends.

Dr. McDowell performed numerous other operations, but of their nature I am not apprised; nor would it be necessary, if I were, to refer to them particularly here. His anatomical knowledge, courage and dexterity were sufficient to enable him to execute any operation that might have been required within the extensive circle of his practice. It cannot be supposed, for a moment, that the man who was the first to excise a diseased ovary, and who cut thirty-two patients for stone without a single failure, would shrink from the performance of any surgical duty, however novel or hazardous, provided he was certain that it was imperatively demanded by the circumstances of the case.

He paid much attention to the subject of hernia. He often operated successfully for the relief of strangulation, and performed many radical cures by means of the truss. His reputation in this branch of surgery attracted patients to him from a great distance. President Polk, early in life, was one of his subjects, and one of those who were permanently cured by his skill.

As a surgeon, Dr. McDowell was exceedingly cautious. He never undertook an operation until his own mind and the patient's system were prepared to his entire satisfaction. Notwithstanding his extraordinary accuracy in anatomical and surgical knowledge, he never operated, in any important case, without carefully reviewing the relations of the structures involved, and referring to the best surgical authorities in his library on the subject. His pupils were obliged to do the same thing, as well as to examine the case, and favor him with their opinion on it. His assistants were carefully selected, and regularly drilled, until, like so many Thespians, they perfectly understood their parts.

He was remarkably kind to his patients, sympathizing with them in their suffering, and encouraging them by tender and soothing expressions. His hand never quivered in an operation; nor did his mind quail; but his face flushed, and even in the depth of winter, the perspiration often started from every pore. Dr. Alban G. Smith, now Dr. Goldsmith, who knew Dr. McDowell intimately, and who is himself an excellent operator, writes me that he was the best operator he ever saw, in all cases where he had a rule to guide him. He always preferred to operate on Sunday mornings, saying that he liked to have the benefit of the prayers of the church.

He was an accomplished anatomist. He made it a business to dissect, more or less, every winter, and he took special pains, on such occasions, to aid his pupils in acquiring a knowledge of the human structure. Subjects were not always obtained, at that period, without trouble and even risk.

Dr. McDowell was no writer. The only contributions he ever made to medical literature are his first five cases of ovariotomy, in the seventh and ninth volumes of the Philadelphia Eclectic Repertory. It is a subject of deep regret that he should have felt, throughout the whole of his life, such a deep repugnance to the publication of the results of his experience. Extensively engaged as he was for so long a period in the practice of medicine and surgery, he must have accumulated a vast amount of knowledge, most valuable to the profession and to suffering humanity, and eminently conducive to the extension of his own fame. But such exercise was distasteful to him, and no remonstrance, on the part of his friends, could induce him to engage in it. Temporary notoriety and posthumous fame were subjects alike of indifference to him. He pursued the "even tenor of his way," and his habits were so confirmed that it was impossible to change them.

Dr. McDowell was an honorary member of several medical associations.

The Medical Society of Philadelphia, one of the oldest and most respectable institutions of the kind in the country, sent him its diploma in 1807. In 1825, he received from the University of Maryland, then in the height of its renown, the honorary degree of Doctor of Medicine—a distinction which was a full acknowledgment of his exalted reputation—and which afforded him genuine gratification, the more especially as it was unsolicited on his part.

Had Dr. McDowell lived in France he would have been elected a member of the Royal Academy of Surgery, received the cross of the Legion of Honor from the king, and obtained a magnificent reward from the government, as an acknowledgment of the services which he rendered his country, his profession, and his fellow-creatures. His own country, and especially his own neighborhood and State, failed to appreciate him. I am told by gentlemen whose veracity is indisputable, that but few of his immediate fellow-citizens were capable of drawing a just distinction between him and the merest charlatans in his vicinity. Such must ever be the fate of true greatness in all new communities. Dr. McDowell had the misfortune to live before his time; he was born in advance of his age.

He was a kind-hearted, amiable man, an urbane and accomplished gentleman, a benevolent physician, a warm and generous friend, an excellent neighbor, an affectionate husband, and an indulgent parent. His character, in all the relations of life, was most exemplary. Of a lively, social temperament, abounding in wit and pleasantry, he was the master-spirit and delight of every company which he honored with his presence. No man was ever more agreeable, more amusing, more unassuming. Frank in his manners, and easy of access, it was impossible to be a stranger in his society, or to leave his presence without a feeling of regret.

As a scholar he was entitled to no ordinary rank in comparison even with some of his most distinguished contemporaries of the learned profession of which he was a member. He was much devoted to study, especially in early life, and was a most admirable recitationist. He was fond of Greek and Latin, a knowledge of which he retained long after his return from Europe. But historical and belles-lettres literature occupied more of his time and attention than classical and scientific works. Burns and Scott were his greatest favorites. In his readings of these authors, he rolled the Scottish idiom upon his tongue in a manner perfectly indescribable. His recitations from Scottish dialogues, adapting his intonations to the supposed character of the speaker, were richer and more exciting than any theatrical exhibition.

He was fond of music, and sung a variety of odes and catches in Latin, English, and Scotch, in good taste and with fine comic effect. His favorite pieces—those of a comic and humorous character—he frequently accompanied with his violin, an instrument to which he was very partial, but upon which he was a poor performer. Like Themistocles, the Athenian, "he could not fiddle, but yet he could make a small town a great city;" he could achieve wonders in surgery, such as had never been achieved before; and thus immortalize his State and country.

His excellence in the Scottish dialect and melody is probably attributable to his summer rambles in Scotland, during the vacations of the medical sessions in the University of Edinburgh. In company with two of his classmates, Dr. Brown and Dr. Speed, of Kentucky, he perambulated a considerable portion of that "land o' cakes," much to their mutual delight and edification. They traveled on foot, each packing a change of clothes in a wallet. In the tour, Dr. McDowell met with several respectable members of his family connection, who recognized and received him as a clansman, pretty much after the style and manner of hospitality commemorated and immortalized by Burns and Scott.

The travelers were well provided with letters to distinguished personages "en route," who never failed to treat them with marked attention and respect. On approaching the residences of these individuals, they always hired a conveyance, and, riding up in due form and style, were received accordingly. They, however, if their entertainment was to their liking, soon "let the cat out of the wallet;" immediately upon which all formality ceased, and they were carried about, all over the neighborhood, either on horseback or in a coach, as they happened to fall in with a commoner or a "gig-man," and exhibited to all sorts of people as gentlemen from the extreme backwoods of America. It is very questionable whether the United States have had, at any time since, the good fortune to be more creditably represented in that ancient and interesting country; a trio of equal intelligence, of fine looks, wit, and good fellowship is rarely to be found anywhere.

After his return to Kentucky, Dr. McDowell frequently recurred, in terms of the greatest delight, to the happy hours spent in these peregrinations, recounting with peculiar glee the incidents which befell him and his backwoods companions. He ever after cherished a warm attachment for the Scotch, their beautiful and romantic country, and their noble scientific and charitable institutions.

His library was quite extensive for the period in which he lived, consisting of all the standard medical works, many of which he had brought from Europe. On the practice of physic he always procured and read the most celebrated authorities; more, says one of his pupils, on account of his students, of whom he always had a considerable number, than of his patients. He was an ardent admirer of Sydenham and Cullen, and never could appreciate any advances worthy of note upon these celebrated writers. With many of his contemporaries, he regarded the portraitures of disease, delineated by the hands of these masters, as inimitable. In his judgment, all other writers on the practice of medicine were mere bunglers and copyists; a decision in which nearly all intelligent professional men at that period concurred. He was in the habit of earnestly cautioning his students against too free a use of medicines. As a *secret*, he apprised them of his impression that the employment of medical drugs was more of a curse than a blessing to the human race, and that quackery perpetrated much more mischief and destruction by their means, than the science of the profession could counteract. In the surgical branch of the profession he took great delight; he characterized it as the *certain* branch of the healing art, and spared no pains to advance and perpetuate his knowledge of it. He does not seem to have had any particular fondness for the practice of medicine, as he always had a partner upon whom devolved most of this kind of business, especially after he had achieved some reputation as a surgeon.

His fees for surgical operations were regulated, as a general rule, by the ability of his patients. As might be supposed, from the extent of his practice, and from his benevolent disposition, he frequently rendered his services gratuitously. Pauper patients, no doubt, often went to him from a great distance, and McDowell would have been the last man to turn a deaf ear to their entreaties for advice and relief. To a humane surgeon, fully appreciating his duty, the claims of the sick poor appeal with irresistible force, proving paramount to every selfish consideration; they are his best subjects, because, in the language of Boerhaave, God is their paymaster, and because the expression of their gratitude is voluntary, not extorted by the hope of obtaining a small bill for services received from their professional attendants.

Occasionally his fees were large: in one instance almost princely. I allude to the case of Mrs. Overton, upon whom he performed the operation of ovariotomy, in the summer of 1822. This lady lived in the vicinity of the Hermitage, in Tennessee, the residence of the late President Jackson, and Dr. McDowell had agreed to operate upon her, at her own house, for five hundred dollars. He remained with her for some days, and on the morning of his departure, her husband, a highly respectable and intelligent citizen, gave him a check, as he supposed, for the stipulated sum, on one of the banks of Nashville. On presenting it, he discovered that it was drawn for fifteen hundred dollars, instead of five hundred. Presuming that a mistake had been made, he immediately dispatched his servant to the gentleman, who replied that no mistake had occurred, and that the services he had received from Dr. McDowell more than counterbalanced the sum he had paid him. Such generous liberality was alike honorable to the giver and to the receiver. So far as I know, this was the largest fee ever paid in this country for a surgical operation. Considering the value of money at that time in the Southwest, or, in other words, the cheapness of living, and the comparatively small compensation for professional services generally, it was fully equivalent to the celebrated fee of a thousand guineas paid by Mr. Hyatt, a West Indian merchant, for an operation performed upon him by Sir Astley Cooper.

Dr. McDowell was not wealthy; his estate at the time of his death was estimated at from forty to fifty thousand dollars. His mode of living was plain and unostentatious; he was always glad to see his friends, and to extend his hospitalities to them.

He was a man of enlarged and liberal views. He spent his money and his time freely upon charitable objects, and manifested great interest in advancing the prosperity of Danville, the scene of his professional labors and renown. Of Centre College, located at that town, and at present the most successful literary institution in Kentucky, he was one of the founders and original trustees; subscribing liberally toward its support.

Dr. McDowell was always a decided Christian in his feelings and conduct. At the time of his settlement at Danville, in the latter part of the last century, nearly the entire male population of the village was atheistically inclined; and not a few were of the Robespierreian school, having achieved the grand discovery that "death is but an eternal sleep." With these men he had no sympathy, though he was of too benevolent and tolerant a nature to fall out with any one for entertaining different tenets from his own.

In 1828 he united himself with the Episcopal Church, and granted a lot at Danville on which was afterwards erected the present church edifice of that town.

Dr. McDowell expired at Danville, on the 25th of June, 1830. His disease was inflammatory fever, terminating his life on the fourteenth day of the attack, in the fifty-ninth year of his age. For him death had no terrors, the grave no victory. He awaited his end with the patience of a Christian, and the calmness of a philosopher. He had "set his house in order," and met death with a serene and composed mind. From the very moment of his seizure he had a presentiment that he should not survive it.

To the professional pilgrim of the West it will not be uninteresting to know that the remains of Kentucky's first great surgeon repose in the family burial-ground of Governor Shelby, five miles from Danville. His tombstone, a plain slab of marble, bears the simple inscription of his name, "Ephraim McDowell."

In person, Dr. McDowell was nearly six feet in height, with a florid complexion, and very black eyes. He was of a remarkably happy disposition, and rather inclined to corpulency. Up to the very time of his sickness he was one of the most active men in Kentucky. As an illustration of his agility and muscular strength, the following anecdote, which he often narrated with special glee, affords a striking example. While he sojourned in Edinburgh, a celebrated Irish foot-racer, a sort of Mike Fink, arrived, boasting that he could out-run, out-hop, and out-jump any man in the city, and bantering the whole medical class. McDowell was selected as their champion. The distance was sixty yards, and the stake ten guineas; the trial took place in the College grounds, and the American purposely allowed himself to be loser. A second race, for one hundred guineas, and at an increased distance, came off soon afterwards, and this time the Irishman, after much bullying, was badly beaten, much to his own chagrin and the gratification of the students.

Dr. McDowell remained faithful to his profession until the last moments of his life. He literally died in the harness. A few months before his final illness, he commenced the building of a large and beautiful mansion in the country, two miles from Danville, where he had intended to spend the evening of his life, away from the cares and fatigues of a busy practice. Death, as has already been seen, frustrated this design. The mansion was finished, but was occupied by other tenants.

